# Effects of telework on anxiety and depression across the United States during the covid-19 crisis

**DOI:** 10.1371/journal.pone.0280156

**Published:** 2023-01-20

**Authors:** Nazmul Islam, Kyle Baun, Rachel Racette

**Affiliations:** 1 Department of Humanities, Bangladesh University of Engineering and Technology (BUET), Dhaka, Bangladesh; 2 Department of Economics, Colgate University, Hamilton, NY, United States of America; UCL: University College London, UNITED KINGDOM

## Abstract

This study serves to provide evidence on how the increase in people working from home due to government induced social distancing measures is contributing to the frequency of individuals suffering from depression or anxiety. Using a compilation of datasets from the NHIS, Household Pulse Survey, and the Oxford Covid-19 Response Tracker, we find a general trend of increased rates of depression and anxiety in those who moved to a remote working format. However, while all regions have an increased frequency in anxiety for those who switched to telework, those in the Northeast and West (that also have implemented strict lockdown measures related to social distancing) have slightly higher rates of anxiety compared to those in the South and Midwest.

## I. Introduction

The World Health Organization (WHO) declared Covid-19 a global pandemic in March 2020, leading governments around the world to take drastic measures to try to curb the spread of the virus. Government responses have varied significantly, with some countries implementing draconian lockdowns (Australia, New Zealand, China), while others had less aggressive interventions (Sweden and USA). Restrictions were implemented at the guidance of epidemiologists with the goal of restricting movement to limit the number of deaths in the medium and short run [1]. Interventions include, but are not limited to, national lockdowns, curfews, closures of schools and non-essential workplaces, and limiting of social gatherings [2]. As a result of these restrictions, people’s day-to-day lives have been considerably disrupted.

Stricter interventions have been proven to be successful with lowering the number of cases but have come at an economic cost. While the cost of these interventions on GDP and labor market outcomes are significant, there are other potential social costs which include disruption to schooling, trust in government, and well-being [3]. As such, a debate has begun over whether the cost of lockdowns and other government mandated restrictions outweigh the benefits of reducing the spread of Covid-19. Poor mental health and a decrease in social well-being have come to the forefront of issues caused by social distancing interventions and are being associated with an increase in anxiety, depression, and stress [4, 5]. Consequently, there has been a demand to investigate the impact of Covid-19 on people’s mental health and psychological well-being [2].

One of the major consequences of the pandemic has been to the US labor market, which has been transformed through a large increase in the prevalence working remotely, also known as teleworking. [6] reports that in April 2020, 31% of employed workers had switched to telework. Variations in teleworking arise among industries and professions due to their different degrees of digitalization. [7] shows that although teleworking rates by industry differ significantly across nations, the trends are usually analogous. Businesses connected with physical production, such as construction, health and social care, accommodation, and food services as well as transport and warehousing had relatively low rates of teleworking during the pandemic. On the other hand, [8] finds businesses that were already extremely digitalized including information and communication services, professional, financial services as well as scientific and technical services achieved much higher rates of teleworking–more than 50% of employees, on average. Public administration, which would be anticipated to “lead by example” in a situation where private sector firms are being persuaded to let workers work from home, too got 50% teleworking, on the whole [8].

Literature examining the increased prevalence of teleworking has determined that individuals may face a number of adversities, including social withdrawal, and lowered self-esteem, all of which can undermine mental health [9].

Research assessing the physical health of workers as a result of telework is ambiguous. [10] shows that the extent of telework is positively associated with employee health. [10] finds that non-teleworkers to be at a significantly higher risk of poor nutrition, physical inactivity, and tobacco use than teleworking workers. Moreover, workers performing 50% or more of their teleworking hours during traditional hours were at a significantly lower risk of alcohol abuse (Henke et al., 2016). In contrast, those who telework during non-traditional hours or over the weekend were at a higher risk of alcohol abuse than both those who telework during traditional hours and non-teleworkers [10]. More recent research conducted during the COVID-19 pandemic [11] also identifies changes in substance use behavior was related to work (e.g., telework) or other factors associated with well-being. Their study finds that individual-level changes in alcohol use in US adults are correlated with negative consequences, from before to during the coronavirus disease 2019 pandemic. According to [12], both men and women had lower systolic blood pressure, a known stress indicator when teleworking versus working physically in the office.

Results from [13] indicate that workers reporting higher levels of flexibility at work also reported a higher number of hours slept on average. Moreover, [14] shows that employees transitioning from the main office to teleworking during a mandated COVID-19 lockdown in Japan reports having more sleep after their transition to home-based teleworking. However, [15] finds though there are proper sleep hygiene behaviors that may help workers’ well-being, sleep duration and quality can also be affected by factors such as presence of children in the home, work schedules, chronic health conditions, and other individual differences.

[16] finds telework may have positive effects on well-being because it might allow people to reconcile work and family, it decreases commuting (which is known to have negative effects on well-being), may permit more time for individuals to meet friends, family and/or go outdoor, and more workplace flexibility, all of which are associated with better mental health. Therefore, there is evidence that telework is related to greater job satisfaction during pre-COVID-19 situation [16, 17]. However, [18] did not find any such effects are found during COVID-19.

[19] notes that prior research has yet to determine how the prevalence of teleworking impacts mental health during Covid-19 at the state-level. [20] suggests that it is necessary to consider how broader, state-level, socio-political contexts shape mental health outcomes related to the pandemic. According to [21] as a result of the devolution of power from the federal government to state level, states’ social and political policies have substantial influence on their population’s health [22]. Despite this, little is known about how state-level policies can mitigate or intensify the mental health costs induced by Covid-19. Therefore, it is imperative to increase our understanding of mental health trends during the current pandemic, especially with regard to the implications of social- distancing policies, as it will enable state-level policy makers to make informed decisions on the current situation, where the overall benefit of relaxing social distancing measures is unclear [3].

The federal government has enabled states to set their own policies with regards to dealing with the pandemic (i.e., social distancing regulations). Across the US, states are implementing differing policies to protect citizens, contain the spread of the virus and boost local economies. For instance, states have the ability to decide whether or not to close schools. A state that has kept schools open may alleviate stress for individuals who have switched to teleworking as they would not have to worry about childcare responsibilities during work hours. Therefore, since policies regarding social distancing measures vary across states, mental health trends will likely also vary across states. As such, we posit those states who have enacted heavier social distancing measures will have a higher percentage of teleworkers as well as a great prevalence of residents suffering from mental health illness than states that have limited social distancing measures. Thus, we hypothesize that adults who have switched to teleworking will face varying degrees of mental health adversity, depending on their region of residency, and state-level policy decisions will have significant influence over mental health effects caused by Covid-19. This study considers the extent to which state policy contexts, with regards to pandemic- specific policies, shape disparities in mental health by teleworking status during the pandemic. This paper aims to examine the following research questions:

- To what extent is teleworking associated with an increased prevalence of mental health illness (i.e., depression and anxiety) during the Covid-19 Pandemic?
- Which gender, age, household income, and level of educational attainment are most likely to experience mental health issues as a result of the increased prevalence of teleworking?
- Do disparities in depression and anxiety vary across regions, given that regional stringency levels related to social distancing measures vary across the US?

In line with prior research [19], this study focuses on anxiety and depression as two unambiguous gauges commonly used to assess mental health during a time of health and economic crisis. With regards to our analysis on the effect of state-level policies on mental health, it should be noted that our study examines regional-level policies, rather than state-level policies. The phrase ‘regional-level policies’ will be used synonymously with ‘regional stringency score’ throughout the paper and captures the overall strictness of social distancing measures for each region. We recognize this as a major limitation to our study, and it is the result of insufficient state-level data in one of our samples.

Finally, this study adds to prior research by considering the psychological toll of the pandemic due to telework, as well as reporting variations of these consequences across regions and regional-level policies implemented in response to the pandemic.

The evidence and knowledge we gain from this study applies to the broader societal implications as the remote work on the rise [23]. The results from [23] show that between 2000 and 2010, people who worked at least one day at home per week increased by over 4 million 35%. The population of occasional distant workers grew from 9.2 million to 13.4 million during this decade [23].

The rest of the paper proceeds as follows. Section II depicts the literature review. Section III presents data description and analysis. Section IV describes empirical models and estimation methods. Section V documents empirical results. Section VI concludes.

## II. Literature review

With the progression of the Covid-19 pandemic has come a sweeping increase in change to remote format working. Telework has implications for increased rates of anxiety and depression, as it has resulted in workers being disconnected from their traditional office network and consequently much of their emotional and social support is lost [24]. A study reports that individuals who depend on others at work tend to have increased negative effects from a change to telework during the Covid-19 pandemic and are more prone to experience conflict at home [25]. [24] defines isolation as “the feeling that one is cut off from others and it occurs when the desire for support, understanding, and other social and emotional aspects of interaction are not met.” This increased isolation is a large contributor to increased frequency in depression among teleworkers [9]. In addition, telework removes an important line between home and work life, leading to a loss of traditional work roles and often resulting in higher workloads. This has led to higher levels of work exhaustion, contributing to the development of stress and anxiety [24].

The response to telework varies by demography. Women are more likely to look upon telework favorably and advocate for its use in the future. A study done in Belgium shows that women are more likely to have the role of caretaker and a career. A move to telework thus is conducive to allowing women to complete both jobs [25]. Older individuals as well, tend to look upon telework more favorably. This can likely be attributed to telework often being completed in a quieter environment in which older individuals may work better in [25].

Social distancing measures and state enacted restrictive measures have resulted in higher levels of depression and anxiety as well, creating the same sort of isolation conditions as telework. A study completed in Germany found that during a week of federally enforced lockdown measures, from March 17th to March 22^nd^ in early 2020, there was a 20% increase in counseling requests spurred by isolation and depression, which then decreased after the week of lockdown was completed [26]. Research completed on the SARS outbreak in 2003 shows that people who underwent a period of quarantine also showed a decline in mental health that lasted for up to three years [27]. [28] shows that symptoms of depression, loneliness and anxiety were maximum soon after the lockdown came into effect. Ultimately, the signs are moved down to some degree, relating to patterns of habituation. Among people for instance women with higher exposure to serious mental health problem during the lockdown, the fraction with high levels of depression, loneliness and anxiety was significantly higher. These groups also unveiled fewer health-promoting behaviors. Less physical activity, more screen time, and more snacking were associated to higher signs of depression, loneliness, and anxiety throughout the time. [28] also finds that changes in health behaviors over time mostly did not predict variations in mental health symptoms.

Mental health illness rates due to lockdowns have also been shown to vary by demographic. Young women (ages 16–24) experience the highest increase in mental health deterioration, with a study in the UK showing an increase in those reporting a severe issue from 17.6% to 35.2% [29]. In contrast, men over the age of 65 undergoing the same study reported little change. While these inequalities are reported to have existed prior to the Covid-19 crisis and the ensuing lockdown, these events have greatly exacerbated the disparities.

[30] found that over 90% of “new teleworkers”, i.e. teleworkers who did not usually work from home before the COVID-19 pandemic, reported being at least as productive at home as they were previously at their usual place of work. This share holds for women and men alike, regardless of age, educational attainment, marital status, parenthood, industry or occupation. The remaining 10% reported accomplishing less work per hour while at home than at their usual workplace due to a lack of interaction with co-workers, family care commitments, inadequate workspace or IT equipment.

On the other hand, [13] finds the positive relationship between telework and healthy behavior along the lines of the empirical literature on flexplace flexibility (as in telework), where employees reporting higher levels of flexibility also reported higher frequencies of physical activity. Correspondingly, [31] shows that greater workplace flexibility (i.e., telework) is associated with less fast-food consumption. Regarding health care utilization, results from [32] indicate that insignificant variations in health care utilization between those with higher and lower flexplace flexibility.

## III. Data description and analysis

This section aims to describe the three different data sources we have used for our study. Moreover, this segment will examine our data through a variety of tables. The primary data source used in this study is from a new national survey administered by the census bureau in conjunction with five federal agencies known as the *Household Pulse Survey* (HPS) 2020 [33]. The HPS has been designed to capture the experiences of individuals throughout the Covid-19 pandemic, with the intention of providing valuable data to policymakers to aid in pandemic recovery. Our sample of data is gathered from October 14, 2020—October 26, 2020. We also use data of total number of Covid-19 cases and total number of deaths due to Covid-19 from the archived of Centers for Disease Control and Prevention (CDC) (2022) [34].

The HPS uses the Census Bureau’s Master Address File (MAF) as the source of sampled housing units (HUs). The sample design was a systematic sample of all eligible HUs, with adjustments utilized to the sampling intervals to choose a big sample for producing state level estimates and estimates for the top 15 Metropolitan Statistical Areas (MSAs). Sixty-six separate sample zones were identified. For each data collection period, independent samples were selected, and each sampled HU was interviewed once. Sample sizes were defined such that a 3-percentage coefficient of variation (CV) for an estimate of 40% of the population would be approached for all sample areas with the exception of the 11 smallest states. In these smaller states, the sample size was decreased to produce a 3.5% CV. The complete sample sizes within the sampling areas were adjusted for a predicted response rate of 9% [35]. Sampled households were contacted by both email if an email was available, and by text if a phone number was available. Contact email addresses and phone numbers were only sent on weekdays and reminders were sent to nonrespondents. The Census Bureau conducted the HPS online using Qualtrics as the data collection platform. Qualtrics is currently used at the Census Bureau for research and development surveys and provides the necessary agility to deploy the HPS quickly and securely.

Respondents of the survey are from all 50 states, with state-specific sample sizes ranging from 774 respondents in Maine to 6395 in California. Evidently, there is an adequate amount of data to determine the prevalence of, and variation of, anxiety and depression across different regions with regards to telework. To examine state-level contexts with regards to their respective social distancing policies, we merged data on individual state’s containment measures (refer to *state context*) with the HPS. The sample used for our study includes only respondents with non-missing information on mental health outcomes. Moreover, we included individuals aged 18–65, as the focus of our study is on telework, and those older than 65 are likely not affected by this. Table 1 provides an overview of our sample from the [33, 35, 36].

**Table 1:** 2020 household pulse survey data.

	Overall		No Change in Work		Started Telework	
2020	Mean/%	(S.D)	Mean/%	(S.D.)	Mean/%	(S.D.)
**Started Telework**	52.49%					
**Depression**	22.62%		24.44%		20.96%	
**Anxiety**	31.72%		31.05%		33.29%	
**Age (range 18–65)**	45.154	12.125	45.15	12.13	45.15	12.13
**Male**	41.07%		39.93%		41.74%	
**Educational Attainment**						
**Less than high school**	1.91%		2.58%		0.54%	
**High school or equivalent**	11.34%		17.10%		4.44%	
**Some college**	21.37%		27.22%		14.99%	
**Associate degree**	10.39%		13.56%		7.36%	
**Bachelor’s degree**	29.56%		23.90%		36.56%	
**Graduate degree**	25.44%		15.62%		36.10%	
**Household Income**						
**<$35,000**	18.13%		26.85%		6.48%	
**$35.000 - $49,999**	10.56%		14.43%		6.28%	
**$50.000-$74.999**	17.40%		20.70%		14.17%	
**$75,000—$99,999**	14.72%		14.72%		15.35%	
**>$100.000**	39.19%		23.31%		57.71%	
**Region**						
**North East**	15.46%		13.84%		17.47%	
**South**	30.80%		31.80%		29.58%	
**Mid-West**	20.74%		22.11%		19.54%	
**West**	32.99%		32.24%		33.40%	

•Percentages calculated using Stata tabulation code

The main weakness of the HPS is that it only began collecting data in April 2020, and therefore this sample is not able to evaluate the impact of Covid-19 on its own. As a result of this limitation, it was necessary to find another data set that collected similar data. The US Census Bureau administers another survey known the *National Health Interview Survey* (NHIS) 2019 [37], which collects many of the same demographic statistics, as well as data regarding depression and anxiety.

The NHIS is a cross-sectional household interview survey. The NHIS universe includes residents of households and noninstitutional group quarters namely homeless shelters, rooming houses, and group homes residing within the 50 states and the District of Columbia at the time of the interview. Persons residing temporarily in student dormitories or temporary housing are sampled within the households that they reside in permanently. Persons excluded from the universe are those with no fixed household address including homeless and transient persons not residing in shelters, active duty military personnel and civilians living on military bases, persons in long-term care institutions (e.g., nursing homes for the elderly, hospitals for the chronically ill or physically or intellectually disabled, and wards for abused or neglected children), persons in correctional facilities (e.g., prisons or jails, juvenile detention centers, and halfway houses), and U.S. nationals living in foreign countries. While active-duty Armed Forces personnel cannot be sampled for inclusion in the survey, any civilians residing with Armed Forces personnel in non-military housing are eligible to be sampled. The household response rate is calculated by dividing the number of interviewed households (33,138) by the sum of the number of interviewed households (33,138) and the number of nonresponding households (21,093). Nonresponding households are eligible households that were not interviewed for a variety of reasons, including language barriers, no one home after repeated contact attempts, refusal, household records rejected for insufficient data, or other reasons for no interview. The total household response rate for NHIS 2019 was 61.1%. An interviewed household is defined as one where the household roster and a substantial portion of either the Sample Adult interview or the Sample Child interview (if one or more children reside in the household) is completed.

Given the parallels between the HPS and the NHIS, we determined that it would be feasible to mesh both samples together, enabling us to have a pre-Covid-19 control group (NHIS), as well as a Covid-19 treatment group (HPS). Moreover, using the HPS and NHIS together follows similar research that assesses the prevalence of anxiety and depression during the 2020 Covid-19 pandemic [38]. Table 2 highlights the main variables of interest from the [37]. Note, the same restrictions were applied to this data set as were applied to the HPS.

**Table 2:** 2019 national health interview survey data.

2019	Mean/%	(S.D)
**Started Telework**	0	
**Depression**	18.87%	
**Anxiety**	15.41%	
**Age (range 18–65)**	43.54	13.50
**Male**	46.04%	
**Educational Attainment**		
**Less than high school**	9.28%	
**High school**	25.77%	
**Some college**	16.39%	
**Associate degree**	13.11%	
**Bachelor’s degree**	21.85%	
**Graduate degree**	13.59%	
**Household Income**		
**< $35,000**	29.41%	
**$35,000-$49,999**	13.02%	
**$50,000-$74,999**	18.07%	
**$75,000-$99,999**	12.47%	
**$100000 or more**	27.04%	
**Region**		
**North-East**	16.91%	
**South**	22.20%	
**Mid-West**	36.49%	
**West**	24.40%	

•Percentages calculated using Stata tabulation code

The HPS and the NHIS samples, when combined, provide us with a data set that contains pre-Covid-19 samples and mid-Covid-19 samples. Given that the HPS has been collecting data following the onset of Covid- 19, it was used to create a time dummy to identify if respondents answered “mid-Covid-19” (= 1). Specifically, this sample collects data from October 14, 2020—October 26, 2020. Respondents of the NHIS sample, responded to the survey in 2019, and are therefore labeled “pre-Covid-19” (= 0). Specifically, this sample collects data from the entire year of 2019.

The HPS and the NHIS are able to capture the frequency of depression and anxiety symptoms for each respondent. To assess anxiety, we use a validated two-item Generalized Anxiety Disorder- 2 (GAD-2) scale [39]. Participants of each survey note how often they feel bothered by (a) feeling nervous, anxious, or on edge and (b) not being able to stop or control worrying over the past 7 days. In both surveys, each of these questions has four possible responses: not at all (= 0); several days (= 1); more than half the days (= 2); Nearly every day (= 3). In order to assess depression, we use the validated two-item Patient Health Questionnaire- 2 (PHQ-2) [40]. Respondents to these surveys report how often they feel bothered by (a) having little interest or pleasure doing things and (b) feeling down, depressed, or hopeless over the past 7 days. Similar to the questions regarding anxiety, these two questions also have four possible responses: not at all (= 0); several days (= 1); more than half the days (= 2); Nearly every day (= 3). In order to obtain the GAD-2 or the PHQ-2, the two responses for anxiety and the two responses for depression are summed respectively for each scale. [39] suggests that a score of 3 or greater on the GAD-2 scale is associated with *generalized anxiety disorder*. Similarly, surveys examining depression suggest that a score of 3 or greater on the PHQ-2 scale can indicate *major depressive disorder* [40]. In line with [19], we use the validated cut point of 3 to create a dichotomous variable for anxiety and depression. For example, a respondent with a score of 3 or greater on the GAD-2 scale (= 1) is determined to have generalized anxiety disorder. Similarly, a respondent with a score of 3 or greater on the PHQ-2 (= 1) is associated with having major depressive disorder.

The variable “Starting to telework as a result of Covid-19” was easily captured in the HPS data with the question “did any adults in this household substitute some or all of their typical in-person work for telework because of the coronavirus pandemic, including yourself?” Respondents were able to select one of three possible options: (1) “Yes, at least one adult substituted some or all of their typical in-person work for telework”; (2) “No, no adults substituted their typical in-person work for telework”; or (3) “No, there has been no change in telework.” Since this study is trying to capture the impact on mental health of individuals who have switched to telework, we combined responses (2) and (3) as these answers imply that there has been no change in their work environment since the onset of the coronavirus pandemic. Thus, if the respondents answered ‘Yes’ to the question, they are reported as shifting to telework and are coded as (= 1); any other response was coded as (= 0). Note, anyone who did not respond was removed from the sample. Moreover, it is necessary to mention that the NHIS sample does not report if people have started teleworking. This was not an issue however since no persons started teleworking due to the onset of the coronavirus pandemic in 2019. Consequently, we reported that all individuals from the NHIS survey were ‘not shifting to telework’ (= 0).

The *State Contexts* refers to the degree of social-distancing restrictions imposed by state- level policy makers. Unfortunately, the HPS was not able to capture this variable on its own as respondents are unable to make an accurate assessment of their respective state’s context. However, the HPS does include the state of residence for each respondent. As a result, our study was able to merge data from the University of Oxford, that uses an index to track policy responses across US states, with the HPS in an effort to evaluate each state’s response to Covid-19. The Oxford Covid-19 Government Response Tracker’s (OxCGRT) US state-level data provides a systematic way to measure and compare US state-level responses to Covid-19. Essentially, the OxCGRT survey combines a variety of data into a single score, which indicates an overall measure of how strict a state’s lockdown measures are. Each state is scored on a scale of 0–100, with 0 indicating ‘no lock down measures’ and 100 indicating ‘the strictest lockdown measures.’

It is necessary to mention that the NHIS sample only indicates which region respondents are from (i.e., Northeast, Midwest etc.) and it does not indicate which state they currently reside. As such, we identify which regions respondents are from, rather than states, in our sample. We used the HPS data to create region categories by combining states into the previously defined categories. Note, Northeast (= 1), South (= 2), Midwest (= 3), and West (= 4). Due to this complication, our sample measures stringency by region opposed to by state. In order to get a stringency score for each region, we created a new variable, “regionstring”, which is associated with the average stringency between all the states in a specific region. Note, all regions have a stringency of ‘0’ if they are associated with the pre Covid-19 time period (given that there were no social distancing measures implemented pre Covid-19).

This study uses several covariates in the models to account for individual sociodemographic characteristics that are likely associated with telework and mental health. Covariates include *age* (in years); *gender* (1 = male); *educational attainment* (less than high school degree (= 1), high school grad (= 2), some college (= 3), associate degree (= 4), bachelor’s degree (= 5), graduate degree (= 6)); *Household Income* (<$35,000 (= 1); $35,000 - $49,999 (= 2); $50,000 - $74,999 (= 3); $75,000 - $99,999 (= 4); $100,000 or more (= 5)). Table 3 below provides a summary of our combined data set, including the dependent variable (depression and anxiety), independent variable (started telework), as well as all other covariates.

**Table 3:** Combined household pulse survey and national health interview survey.

Full Dataset						
	Overall	No change in Work			Started Telework	
All Data	Mean/%	(S.D)	Mean/%	(S.D.)	Mean/%	(S.D.)
**Started Telework**	36.95%					
**Depression**	21.52%		21.50%		20.96%	
**Anxiety**	26.94%		22.86%		33.31%	
**Age (range 18–65)**	44.74%	12.5				
**Male**	42.39%		42.93%		41.74%	
**Educational Attainment**						
**Less than high school**	3.86%		5.86%		0.54%	
**High school or equivalent**	15.15%		21.34%		4.44%	
**Some college**	20.06%		21.93%		14.99%	
**Associate degree**	11.10%		13.34%		7.36%	
**Bachelor’s degree**	27.53%		22.89%		36.56%	
**Graduate degree**	22.31%		14.63%		36.10%	
**Income**						
**< $35,000**	21.69%		28.26%		6.48%	
**$35,000 - $49,999**	11.33%		13.65%		6.28%	
**$50, 000 - $74, 999**	17.61%		19.25%		14.17%	
**$75, 000 - $99, 999**	14.01%		13.48%		15.35%	
**$100,000 or more**	35.36%		25.36%		57.71%	
**Region**						
**North East**	15.81%		15.35%		17.47%	
**South**	32.31%		34.10%		29.58%	
**Mid-West**	21.13%		22.16%		19.54%	
**West**	30.72%		28.41%		33.40%	
**Time**						
**Pre-Covid (2019)**	26.51%					
**During Covid-19 (October 2020)**	73.49%					

*Percentages calculated using Stata tabulation code

## IV. Empirical models and estimation methods

Our dependent variables are GAD (Generalized Anxiety Disorder, often referred to as simply anxiety in our paper) and PHQ (Patient Health Questionnaire): a variable created from a survey measuring depression). We expect that the variable of interest included telework, which has an indirect effect on the probability of having anxiety [GAD] or depression [PHQ]. In addition, other variable of interest was the stringency score of a region, which has a direct effect on rates of telework and an indirect effect on rates of PHQ and GAD. through a direct effect on rates of telework. Our control variables are age, income bracket, attained education, gender, and region of residence which all have indirect effects on GAD and PHQ rates.

In this paper, three models will be run to test our research questions. First, a simple multivariate regression of the entire country to determine the heterogeneous effects of telework across gender, age, income, and level of educational attainment will be run. In addition, we will use a one-way fixed effects model to run a panel data model that allows for fixed effects at the regional level to capture differences in the depression [PHQ] and anxiety [GAD] values associated with each region.

**Fig 1:**
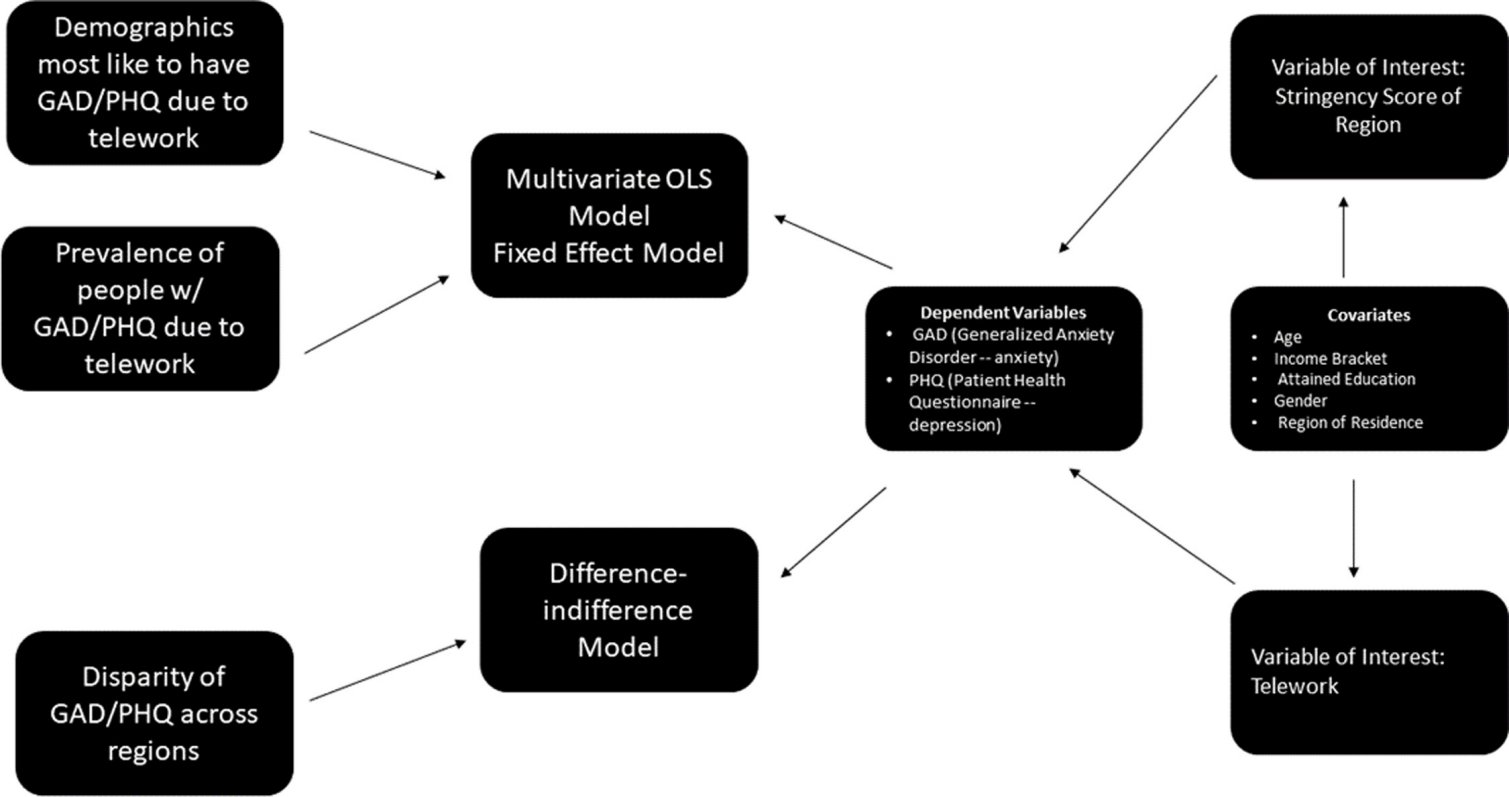
Flow chart illustrating conceptual framework.

Finally, we will run a difference-in-difference model to look at regions with the tightest restrictions (treatment) and with the least restrictions (control) and compare rates of depression [PHQ] and anxiety [GAD]. Fig 1 illustrates a flow chart that conceptualizes our conceptual framework. We used the following models in the paper:

### Multivariate OLS model

We ran a simple multivariate OLS regression to examine the effect of telework on anxiety (GAD) and depression frequency (PHQ):

$$Y_{i}=\beta_{0}+\beta_{1}(tw\_start)+X_{i}\gamma +u_{i}$$

*Y*_*i*_ is the dependent variable that expresses the frequency of GAD or PHQ and *tw_start* is a dummy variable equal to 1 for those who have switched to telework due to Covid-19, and 0 for those who have experienced no change.

The control variables contained within *X*_*i*_*γ* include demographic variables such as age, gender, income bracket, region of habitat, education attained and average region stringency scores. The residual, *u*_*i*_ accounts for the effects of any unincluded variables.

### One-way fixed effects model

We ran a multivariate regression with region fixed effect to examine the differences in rates of depression and anxiety associated with each region:

$$Y_{i}=\beta_{0}+\beta_{1}(tw\_start)+X_{i}\gamma +Region_{i}+v_{i}$$

*Y*_*i*_ is the dependent variable that expresses differences in the depression and anxiety values associated with each region. The dummy variable, *tw_start is* equal to 1 for those who have switched to telework due to Covid-19 and 0 for those who have experienced no change. The remaining control variables contained within *X*_*i*_*γ* include demographic variables such as age, gender, income bracket, region of habitat, and education attained, as well as a year variable that holds the value 2020 or 2019. *Region*_*i*_ is the single fixed effects parameter in the model, and *v*_*i*_ is the random error term.

### Difference-in-difference model

We ran a difference-in-difference model to examine the effect of regional stringency measures on GAD and PHQ:

$$Y_{it}=\beta_{0}+\beta_{1}(region\_01)+\beta_{2}(Treattime)+\beta_{3}(DID)+\beta_{4}(tw\_start)+\beta_{5}X_{it}+\epsilon_{it}$$

*Y*_*it*_ is the dependent variable that expresses the change in GAD and PHQ after a treatment (regional stringency measures) has been applied. *Region_01* is the treatment variable which equals 1 that represents a region that has enacted strong stringency measures for a treatment state and 0 for the control state. The dummy variable, *Treattime* expresses the period after the treatment is performed and takes a value of 1. *DID* is the interaction variable and will equal 1 in the post-treatment time for treated observations, and 0 for all others. *Tw_start* is the main variable of interest, taking values of 1 for those who switched to telework, and 0 for those who experienced no change. *X*_*it*_ contains all other covariates including income bracket, region stringency measures, gender, attained education level, and age. Finally, *ϵ*_*it*_ accounts for any unincluded variables or other error.

## V. Results

**Fig 2:**
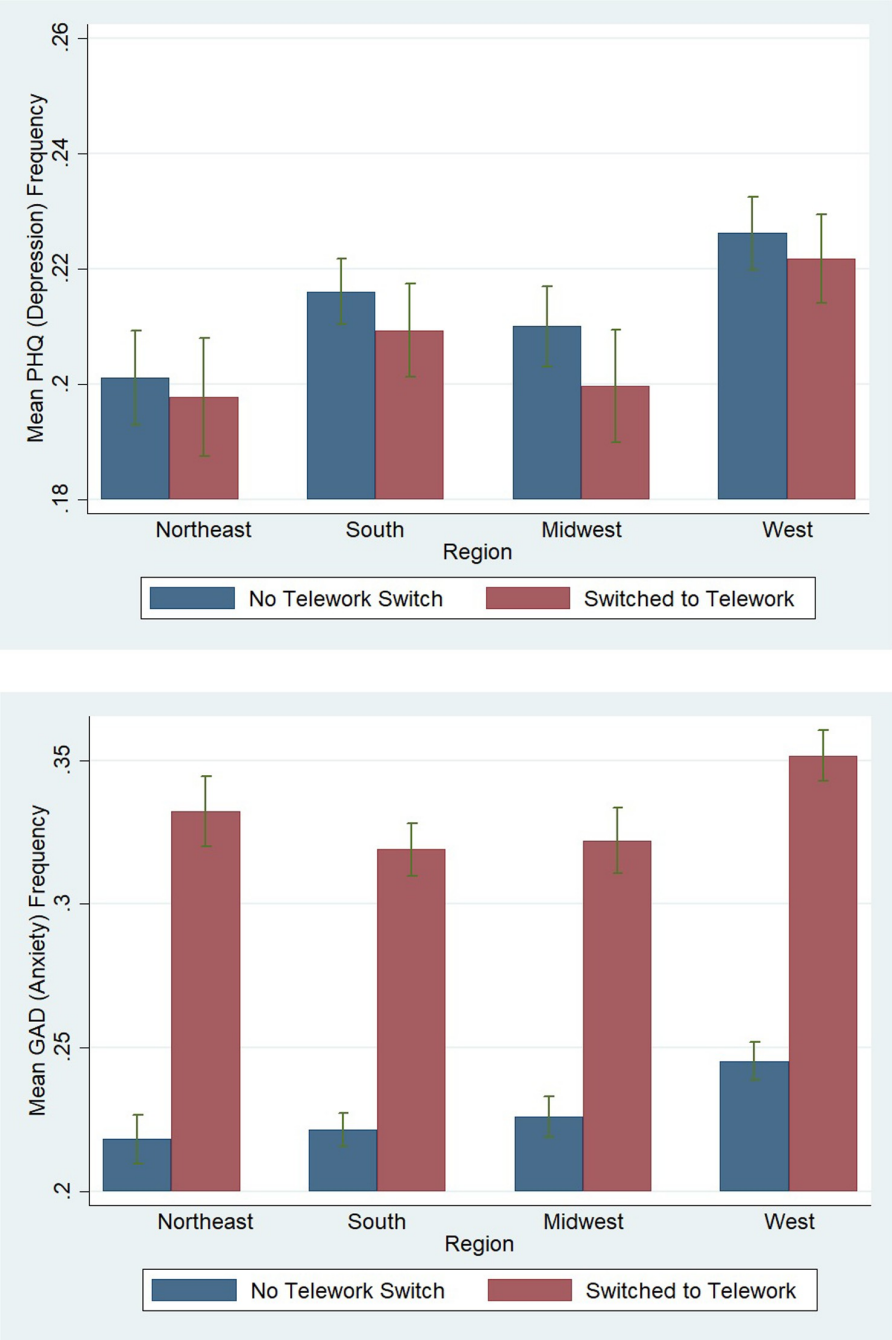
Frequency of PHQ and GAD by region & telework status.

Fig 2A displays a bar graph showing the frequency of people who suffer from depression that have switched to teleworking (due to Covid-19) versus those who have not switched to telework, for each region. Fig 2B displays a bar graph showing the frequency of people who suffer from anxiety that have switched to teleworking versus those who have not switched to telework across various regions.

According to Fig 2A, individuals who have switched to telework see lower rates of depression. Generally, it appears that there is little variation of the rates of depression between the number of people that have switched to telework and those who have not switched to telework in different regions. Fig 2B shows that people who have switched to telework have higher rates of anxiety, nearly 10% greater than those who have not switched to telework. Moreover, while all regions have an increased frequency in anxiety for those who switched to telework, those in the Northeast and West (that also have the toughest stringency measures related to social distancing) have slightly higher rates of anxiety compared to those in the South and Midwest.

Table 4 displays results for three different OLS regressions using the Linear Probability Model (LPM).

**Table 4:** Linear Probability Model (LPM) comparisons.

	Bivariate Regression Model		Multivariate Regression Models			
**Variables**	**Depression**	**Anxiety**	**Depression**	**Anxiety**	**Depression**	**Anxiety**
	**(PHQ)**	**(GAD)**	**(PHQ)**	**(GAD)**	**(PHQ)**	**(GAD)**
	**(I)**	**(II)**	**(III)**	**(IV)**	**(V)**	**(VI)**
**Started teleworking**	-0.00564**	0.104***	-0.0349***	0.0237***	0.00186	0.0320***
	(0.0028)	(0.003)	(0.0033)	(0.0035)	(0.00422)	(0.00453)
**Regional stringency**			0.00132***	0.00363***	0.00202***	0.00436***
			0.0000786	(0.0000835)	(9.90E-05)	(0.000106)
**Current age**					0.00193***	-0.00314***
					(0.000128)	(0.000137)
**Male**					-0.0355***	-0.0840***
					(0.00317)	(0.0034)
**Household Income ($35,000 - $49,999)**					-0.0848***	-0.0661***
					(0.00609)	(0.00653)
**Household Income ($50,000 - $74,999)**					-0.124***	-0.110***
					(0.00534)	(0.00573)
**Household Income ($75,000 - $99,999)**					-0.162***	-0.139***
					(0.00567)	(0.00609)
**Household Income ($100,000 or more)**					-0.209***	-0.188***
					(0.00494)	(0.00531)
**High school graduate**					-0.0244***	-0.0125
					(0.00931)	(0.00998)
**Some college, no degree**					0.00746	0.00875
					(0.00925)	(0.00992)
**Associate degree**					-0.011	-0.00306
					(0.00968)	(0.0104)
**Bachelor’s degree**					-0.0359***	-0.0108
					(0.00919)	(0.00986)
**Graduate degree**					-0.0510***	-0.00782
					(0.00948)	(0.0102)
**Constant**	0.215***	0.229***	0.189***	0.157***	0.426***	0.451***
	(0.00168)	(0.0018)	(0.00229)	(0.00243)	(0.011)	(0.0118)
**Observations**	92920	92941	92920	92941	69,255	69,251
**R-squared**	0	0.013	0.003	0.032	0.052	0.077

Standard errors are in parentheses.

*** p<0.01,

** p<0.05,

* p<0.1.

The first regression attempts to estimate the relationship between the dependent variables PHQ/GAD and our main independent variable of interest, “start telework” (tw_start). According to this bivariate regression, individuals who switched to telework due to Covid-19 are 0.56% less likely to be depressed than individuals who did not switch. This aligns with our bar graph, which also shows that individuals who switched to telework are slightly less likely to be depressed. Our results related to GAD are also analogous to our bar graph, stating that people who have switched to telework are 10.4% more likely to suffer from depression. Both results are statistically significant at the 1% level.

These results, however, are unlikely to tell the whole story, as there could be other factors influencing the rate of depression and anxiety. In order to account for these other variables, a third multivariate regression attempts to account for these influences. Interestingly, when accounting for all variables and holding them constant, the individuals who switched to telework due to Covid-19 are 0.19% more likely to be depressed than individuals who did not switch. While this interpretation is not statistically significant, it is noteworthy, in that depression increases amongst those who switched to telework, versus decreasing depression (which happened in the first regression). Finally, when interpreting the coefficient of telework in the multivariate regression as it is associated with GAD, we notice that it decreases noticeably compared to the bivariate regression. That is, when holding all else constant, we see that those who have switched to telework due to Covid-19 are 3.2% more likely to suffer from anxiety than those who have not switched to telework. This is much lower than the 10.4% increased likelihood of anxiety for those who switched to telework that was estimated in the bivariate model.

**Fig 3:**
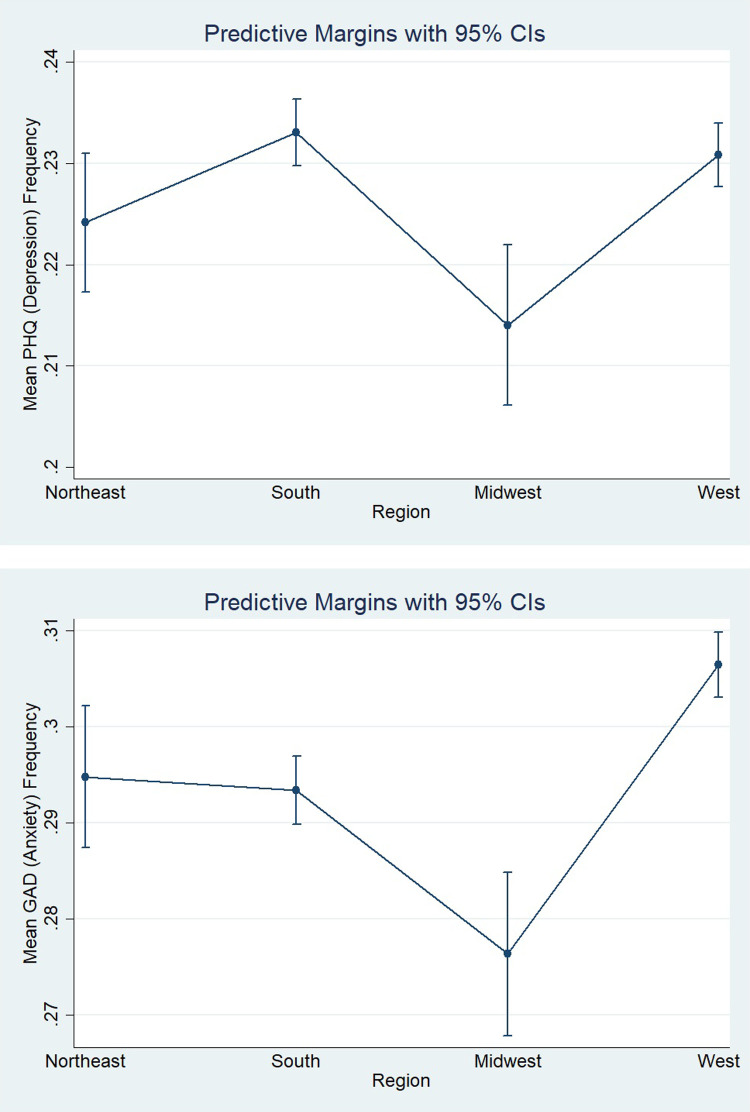
Multivariate Fixed Effect (FE) model.

Fig 3A displays margin plots for depression and Fig 3B shows anxiety, highlighting the frequency of people who suffer from either of these mental illnesses across various regions, taking into consideration, stringency measurements of each region, whether individuals switched to telework, as well as a host of other covariates. According to Fig 3A, people from Midwest have the lowest frequency of depression, while people from the West and the South have the highest rates of depression. Fig 3B implies that people in the Midwest have the lowest frequency of anxiety, while people in the West are the most likely to suffer from anxiety. Finally, when comparing the graphs, it is evident that people are more likely to suffer from anxiety than depression.

Table 5 displays the results of the regression of the fixed effect model and the multivariate OLS regression (from Table 4) using the LPM. Table 5 shows that the results from the fixed effect model are similar to the results from the multivariate regression, suggesting that our results are robust.

**Table 5:** Fixed effect and multivariate OLS model comparison.

	Fixed Effects Models		Multivariate Regression Models	
			(Using LPM Mentioned in Table 4)	

**Variables**	**Depression**	**Anxiety**	**Depression**	**Anxiety**
	**(PHQ)**	**(GAD)**	**(PHQ)**	**(GAD)**
	**(I)**	**(II)**	**(III)**	**(IV)**
**Started teleworking**	0.00211	0.0317***	0.00186	0.0320***
	(0.00423)	(0.00454)	(0.00422)	(0.00453)
**Regional stringency**	0.00284***	0.00327***	0.00202***	0.00436***
	(0.0007)	(0.000751)	(9.90E-05)	(0.000106)
**Current age**	-0.00193***	-0.00314***	-0.00193***	-0.00314***
	(0.000128)	(0.000137)	(0.000128)	(0.000137)
**Male**	-0.0356***	-0.0839***	-0.0355***	-0.0840***
	(0.00317)	(0.0034)	(0.00317)	(0.0034)
**Household Income ($35,000 - $49,999)**	-0.0848***	-0.0661***	-0.0848***	-0.0661***
	(0.00609)	(0.00653)	(0.00609)	(0.00653)
**Household Income ($50,000 - $74,999)**	-0.124***	-0.110***	-0.124***	-0.110***
	(0.00534)	(0.00573)	(0.00534)	(0.00573)
**Household Income ($75,000 - $99,999)**	-0.162***	-0.139***	-0.162***	-0.139***
	(0.00567)	(0.00609)	(0.00567)	(0.00609)
**Household Income ($100,000 or more)**	-0.210***	-0.188***	-0.209***	-0.188***
	(0.00494)	(0.00531)	(0.00494)	(0.00531)
**High school graduate**	-0.0245***	-0.0124	-0.0244***	-0.0125
	(0.00931)	(0.00998)	(0.00931)	(0.00998)
**Some college, no degree**	0.00752	0.00866	0.00746	0.00875
	(0.00925)	(0.00992)	(0.00925)	(0.00992)
**Associate degree**	-0.011	(0.00315	-0.011	-0.00306
	(0.00968)	(0.0104)	(0.00968)	(0.0104)
**Bachelor’s degree**	-0.0358***	-0.0109	-0.0359***	-0.0108
	(0.00919)	(0.00986)	(0.00919)	(0.00986)
**Graduate degree**	-0.0509***	-0.00788	-0.0510***	-0.00782
	(0.00948)	(0.0102)	(0.00948)	(0.0102)
**Time dummy**	-0.0349	0.0464		
	(0.0295)	(0.0316)		
**Constant**	0.434***	0.460***	0.426***	0.451***
	(0.0103)	(0.011)	(0.011)	(0.0118)
**Observations**	69,255	69,251	69,255	69,251
**Number of Regions**	4	4	-	-
**Region Fixed Effects**	Yes	Yes	No	No
**R-squared**	0.052	0.076	0.052	0.077

Standard errors are in parentheses.

*** p<0.01,

** p<0.05,

* p<0.1.

“egender” is a categorical variable and it has two categories. egender = 1 for Male and egender = 0 for Female. Similarly other categorical variables are “income” (5 categories), “eeduc” (“eeduc” = education has six categories) and “region” (4 categories).

As such, both models posit that a 1 unit increase on the regional stringency scale is associated with about 0.2% increase in depression. Similarly, both models represents that a 1 unit increase on the regional stringency scale is associated with about 0.33% (FE model) or about 0.44% (Multivariate) increase in anxiety. With regards to age, both models state that a 1-year increase in age is associated with a 0.19% decrease in depression. Similarly, both models state that a 1-year increase in age is associated with a 0.31% decrease in anxiety. Both models imply that males are 3.6% less likely to suffer from depression than females. Similarly, both models state that males are 8.4% less likely to suffer from anxiety in comparison to females. With regards to income, both models find that as household income increases, depression and anxiety decrease. A similar pattern is seen with regards to educational attainment. In other words, the higher degree a person attains, the less likely they are to suffer from depression or anxiety. Interestingly, there is one anomaly, which is verified by both models, that people with “some college” are more likely to suffer from depression or anxiety compared to all others.

**Fig 4:**
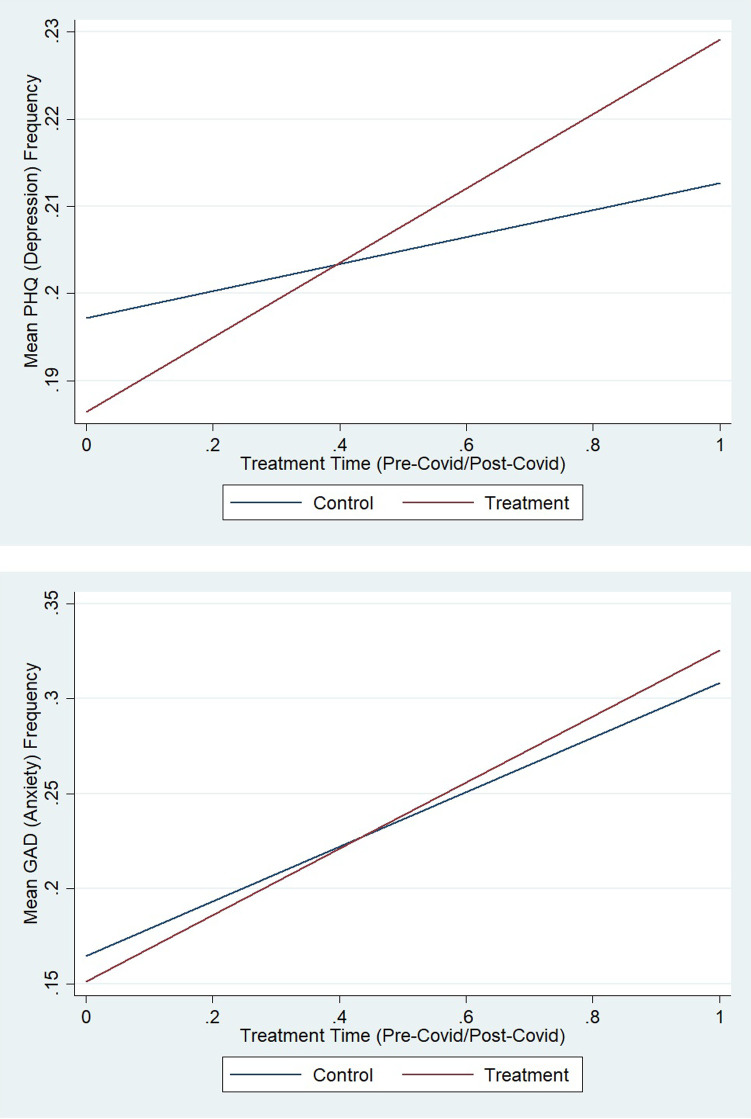
Difference in difference model.

As previously mentioned, unlike the LPM (fixed effect and multivariate) models which assessed the relationship between telework and depression and anxiety, the Difference-in- Difference (DiD) model captures the impact of stringency measures on depression and anxiety during Covid-19. As such, Fig 4 illustrates two DiD models, highlighting the impact of stringency measures on depression in Fig 4A and anxiety in Fig 4B. Fig 4A shows that the control group was more depressed than the treatment group prior to the implementation of strict social distancing measures due to Covid-19. However, following the onset of stringency measures, it appears that those who face the toughest social distancing restrictions (treatment) were more depressed than those who faced only mild social distancing restrictions. When assessing the association between anxiety and stringency measures, we see analogous results in Fig 4B.

Following the implementation of lockdown measures, those who faced the most severe measures (the treatment group) saw an increased prevalence of anxiety compared to those who only faced lenient measures.

The DiD model I in Table 6 shows that the implementation of aggressive stringency measures leads to an increase in depression of 2.71%.

**Table 6:** DiD models with PHQ (depression) as the dependent variable.

Variables	(I)	(II)	(III)	(IV)
**Treat time**	0.0155**	0.0937***	0.135***	0.121***
	(0.00607)	(0.0317)	(0.0344)	(0.0329)
**Region_01**	-0.0107*	-0.0190***	-0.0239***	-0.0187***
	(0.00556)	(0.00653)	(0.00673)	(0.00653)
**_Diff**	0.0271***	0.0543***	0.0658***	0.0614***
	(0.00686)	(0.0123)	(0.0129)	(0.0125)
**Income**		-0.0498***	-0.0500***	-0.0500***
		(0.00116)	(0.00116)	(0.00116)
**Region string**		-0.00114	-0.00236**	-0.00193**
		(0.000915)	(0.000998)	(0.000951)
**Male**		-0.0360***	-0.0361***	-0.0361***
		(0.00317)	(0.00317)	(0.00317)
**Education**		-0.0107***	-0.0107***	-0.0107***
		(0.0012)	(0.0012)	(0.0012)
**Telework start**		0.000611	0.000588	0.000572
		(0.0042)	(0.0042)	(0.0042)
**Current age**		-0.00195***	-0.00195***	-0.00195***
		(0.000127)	(0.000127)	(0.000127)
**Total cases**			-6.39e-08***	
			(2.08E-08)	
**Total deaths**				-3.21e-06***
				(1.05E-06)
**Constant**	0.197***	0.502***	0.521***	0.512***
	(0.0049)	(0.00916)	(0.0111)	(0.00968)
**Observations**	94,217	69,219	69,219	69,219
**R-squared**	0.002	0.051	0.051	0.051

Standard errors are in parentheses.

*** p<0.01,

** p<0.05,

* p<0.1.

When examining the DiD model I in Table 7, we see that aggressive stringency measures are associated with a 3.06% increase in anxiety.

**Table 7:** DiD model with GAD (anxiety) as the dependent variable.

Variables	(I)	(II)	(III)	(IV)
**Treat time**	0.144***	0.128***	0.212***	0.182***
	(0.00645)	(0.034)	(0.0369)	(0.0353)
**Region_01**	-0.0136**	(0.0231***	-0.0331***	-0.0225***
	(0.00591)	-0.00701)	(0.00722)	(0.00701)
**_Diff**	0.0306***	0.0410***	0.0645***	0.0555***
	(0.00729)	(0.0132)	(0.0138)	(0.0134)
**Income**		-0.0449***	-0.0452***	-0.0452***
		(0.00124)	(0.00124)	(0.00124)
**Region string**		0.000643	-0.00184*	-0.00097
		(0.000982)	(0.00107)	(0.00102)
**Male**		-0.0840***	-0.0841***	-0.0841***
		(0.0034)	(0.0034)	(0.0034)
**Education**		-0.00192	-0.00194	-0.00192
		(0.00128)	(0.00128)	(0.00128)
**Telework start**		0.0316***	0.0315***	0.0315***
		(0.00451)	(0.00451)	(0.00451)
**Current age**		-0.00316***	-0.00315***	-0.00314***
		(0.000136)	(0.000136)	(0.000136)
**Total cases**			-1.30e-07***	
			(2.23E-08)	
**Total deaths**				-6.53e-06***
				(1.13E-06)
**Constant**	0.165***	0.515***	0.554***	0.534***
	(0.0052)	(0.00983)	(0.0119)	(0.0104)
**Observations**	94,237	69,215	69,215	69,215
**R-squared**	0.032	0.076	0.077	0.077

Standard errors are in parentheses.

*** p<0.01,

** p<0.05,

* p<0.1.

It is important to note that both of these findings are significant at the 1% level. Model II in Table 6 depicts that the prevalence of depression associated with aggressive social distancing measures increases to 5.43%. Similarly, when model II in Table 7 is applied to determine the frequency of anxiety between the treatment and the control, it appears that strict lockdown measures increase the rates of anxiety by 4.1% in comparison to those who face soft social distancing restrictions.

If we include total number of Covid-19 cases as a control variable in model III in Table 6, we find that the stringency measures lead to an increase in depression of 6.58%. If we add total number of deaths due to Covid-19 as an independent variable, we observe that the stringency measures lead to an increase in depression of 6.14%.

In Table 7, we find that there are higher rates of anxiety if we include total number of Covid-19 cases or total number of deaths due to Covid-19 in model III and model IV respectively compared to models I and II.

## VI. Summary and conclusions

The Covid-19 crisis has resulted in a sharp increase of jobs moving to a remote format. This unprecedented shift in work style varies across regions of the United States, with differing stringency policies prompting differing rates of telework. Using data from three separate national surveys, we have assembled a data set that describes the relationship between telework and rates of depression or General Anxiety Disorder (GAD) across the United States. Regions with higher rates of individuals working remotely tend to correlate with higher rates of anxiety and depression compared to other regions. For example, the [33] shows the West has the highest percentage of individuals who changed to remote work. Correspondingly, the multivariate bar graph in Fig 2 shows that the West has the highest percentage of workers who made the switch to telework and are also suffering from depression or GAD. Conversely, the Midwest has the lowest percentage of individuals who switched to working remotely, and this region contains the lowest frequency of individuals suffering from depression or GAD.

Controlling for regional stringency rates tends to strengthen the relationship between rates of GAD and depression as well. This can be seen in the DiD model which, without a region stringency covariate, shows a 2.71% and 3.06% increase in GAD and depression, respectively, while the DiD model that controls for regional stringency measures, shows a 4.1% increase in the number of individuals suffering from depression, and a 5.4% increase in individuals with GAD. The results are supported by [2] that shows that mental health issues and decreases in social well-being have been shown to be on the rise due to social distancing measures which have increased rates of anxiety and depression.

There is a noticeable disparity in individuals suffering from GAD and depression between regions that received increased stringency measures after the time treatment and the regions that did not.

The rate of individuals suffering from GAD or depression varies by demographic factors as well. Taking region as a fixed effect into account shows that males are less likely to experience anxiety than women. Our findings show that a 1-year increase in age is associated with a 0.31% decrease in anxiety and that males are 8.4% less likely to suffer from anxiety than females. This aligns with previous findings which note that young women have the highest rates of mental health deterioration in contrast to men over 65 years old who have the lowest change [29].

Increasing age also shows a negative correlation to GAD. Income and educational attainment are also significant covariates, as increasing income brackets levels of educational attainment show a decrease in likelihood of suffering from GAD. The same trends for the main variable of interest, telework, and the covariates are present in the individuals suffering from depression.

Our study shows that individuals who have switched to telework and are living in states with stronger lockdown measures are more prone to suffering from anxiety. Our OLS multivariate model shows that all regions have an increased frequency in anxiety for those who switched to telework, but those in the Northeast and the West have slightly higher rates of anxiety compared to those in the South and the Midwest.

This higher frequency in individuals with GAD correlates to the regions with the toughest social distancing stringency measures. We found that the populations of regions with lower stringency measures, such as the Midwest, suffered significantly lower rates of anxiety than the population of regions with higher stringency measures, such as the West.

In contrast, the population which switched to telework shows no difference in the rate of depression compared to the population which had no change. There is little variation in depression rates among regions and once again, the Midwest shows the lowest rates of all the regions.

The sudden isolation and loss of job network due to telework is a shock that has affected a large number of people across the United States. This study is one of the first to explore the relationship between telework and rates of individuals suffering from anxiety or depression, based on regional social distancing restrictions.

This study is a comprehensive documentation on the population of workers who have begun teleworking and the resulting effects on their mental health. Nevertheless, there are limitations to our study. Firstly, the abruptness of Covid-19 means that data concerning the population working remotely prior to the implementation of stringency measures is fairly limited. Secondly, the 2019 NHIS had observations only at the regional level (rather than the state level), so the comparison across the United States is less specific than desired. Thirdly, our sample does not account for industry or labor data, which also could have an effect on the rate of telework. Finally, the Covid-19 crisis is still in progress, so the long-term effects of telework on rates of depression and GAD are still unrevealed.

Our research shows that policymakers would be wise to consider the impact of implementing social distancing measures on the prevalence of telework, and the resulting influence on the mental health crisis. [3] shows that in the UK a variety of factors experienced during lockdowns lead to compounding effects on loss of wellbeing. Continued analysis on the benefits and costs of more stringent measures should be done in order to monitor the effects of telework on levels of depression and anxiety. Officials considering social distancing or lockdown measures should account for the implications they may have on the workforce and the subsequent mental health effects of these policies.

We could better explain and justify our research design if we would consider occupation-based differences in tele workability of jobs. Because, most importantly, the data set is not an individual level panel data. Therefore, claims like “people who switched to telework have higher rates of anxiety” are of course true, but tell us little about the causal direction. It is possible (and likely) that if there is some freedom to choose whether to switch to remote work or not, more anxious people are more likely to switch to remote work. Moreover, we have some doubts that the control variables (education and income) already capture occupation-based differences in tele workability of jobs. So, the difference in mental health between those who switched to remote work and those who continued to work on site, might of course be the result of some jobs being more tele workable and others not. If the tele workability of occupations is then related to anxiety or depression, the correlations of this study would be spurious. This issue may be addressed empirically in future research.
